# Modeling electrical stimulation of retinal ganglion cell with optimizing additive noises for reducing threshold and energy consumption

**DOI:** 10.1186/s12938-017-0333-z

**Published:** 2017-03-27

**Authors:** Jing Wu, Menghua Jin, Qingli Qiao

**Affiliations:** School of Biomedical Engineering & Technology, Tianjin Medical University, Tianjin, 300070 China

**Keywords:** The RGC model, Stimulation thresholds, Energy consumption, Subthreshold signal, Stochastic biphasic pulse sequences, Epiretinal prosthesis

## Abstract

**Background:**

Epiretinal prosthesis is one device for the treatment of blindness, which target retinal ganglion cells (RGCs) by electrodes on retinal surface. The stimulating current of epiretinal prosthesis is an important factor that influences the safety threshold and visual perception. Stochastic resonance (SR) can be used to enhance the detection and transmission of subthreshold stimuli in neurons. Here, it was assumed that SR was a potential way to improve the performance of epiretinal prosthesis. The effect of noises on the response of RGCs to electrical stimulation and the energy of stimulating current was studied based on a RGC model.

**Methods:**

The RGC was modeled as a multi-compartment model consisting of dendrites and its branches, soma and axon. To evoke SR, a subthreshold signal, a series of bipolar rectangular pulse sequences, plus stochastic biphasic pulse sequences as noises, were used as a stimulus to the model. The SR-type behavior in the model was characterized by a “power norm” measure. To decrease energy consumption of the stimulation waveform, the stochastic biphasic pulse sequences were only added to the cathode and anode phase of the subthreshold pulse and the noise parameters were optimized by using a genetic algorithm (GA).

**Results:**

When certain intensity of noise is added to the subthreshold signal, RGC model can fire. With the noise’s RMS amplitudes increased, more spikes were elicited and the curve of power norm presents the inverted U-like graph. The larger pulse width of stochastic biphasic pulse sequences resulted in higher power norm. The energy consumption and charges of the single bipolar rectangular pulse without noise in threshold level are 468.18 pJ, 15.30 nC, and after adding optimized parameters’s noise to the subthreshold signal, they became 314.8174 pJ, 11.9281 nC and were reduced by 32.8 and 22.0%, respectively.

**Conclusions:**

The SR exists in the RGC model and can enhance the representation of RGC model to the subthreshold signal. Adding the stochastic biphasic pulse sequences to the cathode and anode phase of the subthreshold signal helps to reduce stimulation threshold, energy consumption and charge of RGC stimulation. These may be helpful for improving the performance of epiretinal prosthesis.

## Background

Retinitis pigmentosa (RP) and age-related macular degeneration (AMD) lead to blindness in hundreds of thousands of people each year due to the loss of photoreceptors [1, 2]. There is no cure for both diseases. These patients are blind, but there are remaining healthy retinal ganglion cells (RGCs) that can carry retinal inputs to the brain [3]. Epiretinal prosthesis is a kind of artificial organ that restores functional vision to the blind by electrically stimulating surviving retinal neurons with an electrode array implanted on top of the retina [4].

Epiretinal prosthesis such as Argus II has been widely applied to experimental studies and clinical application [5, 6], but many problems still exist in epiretinal prosthesis. For instance, the accuracy of image processing, the biocompatibility and life of the device and visual resolution and field all need to be improved [6]. In image processing, a breakthrough in the encoding and translation of video images into recognizable visual forms has been reported [7]. As well, visual resolution and field can be improved by increasing the number of electrodes in the microelectrode array [8]. However, with the number of electrodes in the microelectrode array increased, the energy consumption of epiretinal prosthesis will also increase, which may lead to excessive heat or nerve damage [9]. Therefore, reducing the energy consumption of epiretinal prosthesis is required.

Many methods of reducing energy consumption have been proposed. Stimulation thresholds have an effect on the energy consumption of epiretinal prosthesis. Low stimulation threshold means that subthreshold stimulus signal can achieve the same efficacy as that of threshold signal. Reducing stimulation thresholds can decrease the energy consumption of epiretinal prosthesis [10]. One approach for reducing threshold is to minimize the distance between the multielectrode array and retina [11]. However, minimizing the distance between the multielectrode array and retina requires superb surgical techniques. Another is to modify the stimulus pulse shape. Many stimulus waveforms, such as sine, triangular, linear, staircase, had been studied in nerve electrical stimulation [12], but changing stimulus shapes increases the difficulty of circuit implementation. Rectangular charge-balanced stimuli are the classical stimulus waveforms applied to the retinal prosthesis. Optimizing these parameters can increase the efficacy of stimulating RGC. A large number of neural stimulation devices that use square stimulation pulses are approved for use in humans and existing devices also make these pulses easy to implement. A method for reducing stimulation threshold of epiretinal prosthesis without changing the biphasic rectangular pulse is expected to be found.

Stochastic resonance (SR) is a phenomenon in which the detection of a subthreshold signal is improved in a nonlinear system by the addition of noise [13]. In the nervous system, neurons are always in a noisy environment and the electrical activity of neurons has a nonlinear threshold characteristic, therefore, the nervous system has the conditions to generate SR. Douglass et al. found SR phenomenon existing in the nervous system, crayfish mechanoreceptors [14]. SR also has been observed in other sensory and central nervous system. It could be utilized to enhance the detection and transmission of weak stimuli in sensory neurons and CA1 hippocampal cells and improve visual motion discrimination [15–17]. SR is also applied to the prosthesis. It is used to improve human balance control and somatosensation [18, 19]. The application of SR in cochlear implant stimulation strategies and enhancing auditory information processing has been studied [20, 21]. In addition, SR is introduced into the operation of the artificial ventilator [22].

In the paper, it was assumed that SR could be used to improve the detection ability of RGC and reduce epiretinal prosthesis stimulation thresholds. The responses of RGC to the subthreshold signal with noises, the influence of noise parameters on energy consumption and optimization of noise parameters will be investigated. The RGC is modeled as a multi-compartment RGC model of extracellular stimulation. A series of biphasic rectangular pulse sequences is used as the model’s subthreshold signal. For the RGC, the possible biological noise sources stem from synaptic input from bipolar cells and voltage-gated ion channels [23]. Here, an artificial noise is applied. It was found that stochastic rectangular pulse sequences could be used to generate SR and are more effective than traditional broadband noise [24], so they are used as noises to generate SR. A “power norm” was used to characterize SR-type behavior. However, the additional noises necessary for generating SR will result in additional energy consumption. In order to reduce the adverse effects of noise, the noises are only added to the cathode and anode phase of the pulses and the noise parameters was optimized by using a genetic algorithm (GA). The GA is a method for solving optimization problems through a process based on the principles of biological evolution. It has been used in the numerical optimization [25] and neural stimulation optimization, such as finding energy-efficient waveform shapes for neural stimulation [26] and optimizing nerve cuff stimulation of targeted regions through use of GA [27].

## Methods

### RGC model

**Fig. 1:**
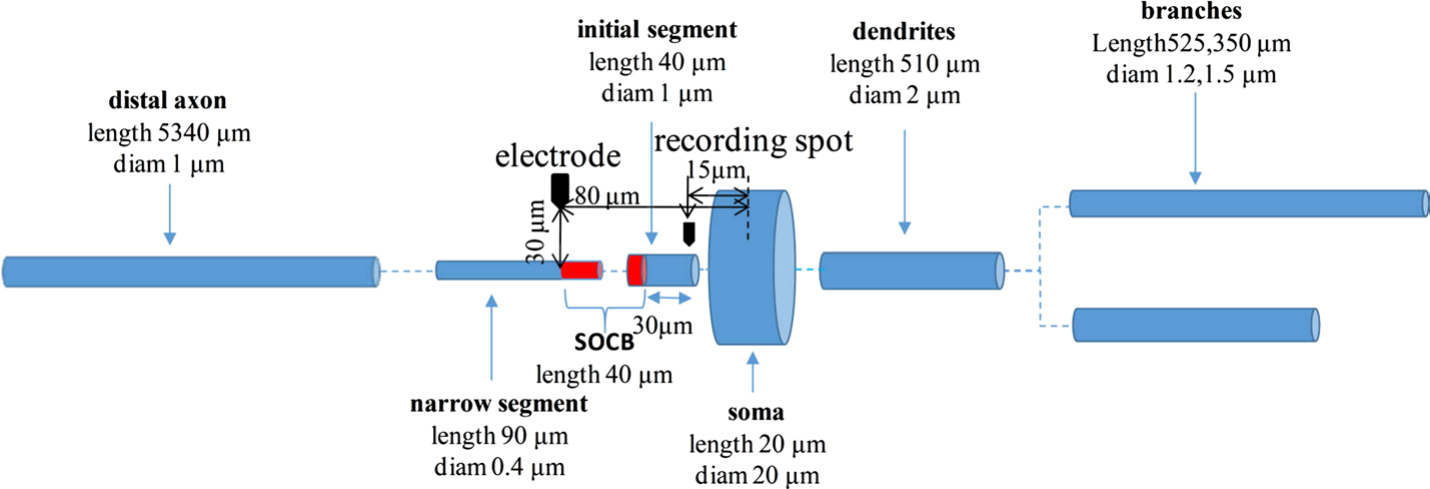
A schematic diagram of the cylinder model of RGC. Cylindrical elements represent compartments. The place of the stimulating electrode and recording spot are indicated by *black arrows*

RGC was simulated as a multi-compartment model of extracellular stimulation with certain geometric and electrical parameters. The cell model was divided into compartments representing the dendrites, soma and axon. The complex dendrites were equivalent to a trunk and two branches. The axon was comprised of four regions: the initial segment, sodium channel band (SOCB), narrow segment, and distal axon. The shapes of the segments were approximated by cylinder and every compartment had its individual geometric and electrical parameters [28]. Each compartment was modeled as a 10-μm-long cylinder except longer dendritic branch which was 15-μm-long [29]. A schematic diagram of the cylinder model of RGC and the diameter and length of each segment [29, 30] are illustrated in Fig. 1.

In the *n*th compartment, applying Kirchoff’s law results in the following equation [28],

1 $$\begin{aligned} C_{m} \frac{{dV_{n} }}{dt} &= - I_{ion,n} + \frac{{V_{n - 1} - V_{n} }}{{R_{n - 1} /2 + R_{n} /2}} + \frac{{V_{n + 1} - V_{n} }}{{R_{n + 1} /2 + R_{n} /2}} \\ & \quad+ \frac{{V_{e,n - 1} - V_{e,n} }}{{R_{n - 1} /2 + R_{n} /2}} + \frac{{V_{e,n + 1} - V_{e,n} }}{{R_{n + 1} /2 + R_{n} /2}} \end{aligned}$$

where *V*_*n*_ is the membrane potential in the *n*th compartment, *C*_*m*_ indicates the specific capacitance of the membrane which is equal to 1 μF/cm^2^, *R* represents the internal resistance of the compartment, *V*_*e*_ is the extracellular potential.

The equation for the extracellular potential is [28]

2 $$V_{e} = \frac{{\rho_{e} I}}{4\pi r}$$

where $\rho_{e} = 1/(60\;\varOmega \;{\text{cm}})$, *r* is the distance from the center of each compartment to the position of the stimulating electrode. $I = I_{s} + I_{n}$ ,is the stimulating current. *I*_*s*_ is the subthreshold signal and *I*_*n*_ is the noise current applied to generate SR.

The membrane properties are described using Fohlmeister–Colman–Miller (FCM) model. The FCM model includes sodium, calcium, delayed rectifier potassium, A-type potassium, Ca-activated potassium and leak conductance [31]. In this paper, sodium persistent, hyperpolarization-activated and T-type low voltage activated (LVA) calcium conductances are added to this RGC model. The ion currents, $I_{ion}$, is calculated as follows [30]

3 $$\begin{aligned} I_{ion,n} &= \overline{g}_{L} (V_{n} - V_{L} ) + \overline{g}_{Na} m^{3} h(V_{n} - V_{Na} ) + \overline{g}_{Ca} c^{3} (V_{n} - V_{Ca} ) \\ & \quad + (\overline{g}_{K} n^{4} + \overline{g}_{K,A} a^{3} h_{A} + \overline{g}_{K,Ca} )(V_{n} - V_{K} ) \\ & \quad + \overline{g}_{h} l(V_{n} - V_{h} ) + \overline{g}_{T} m_{T}^{3} h_{T} \times (V_{n} - V_{T} ) + \overline{g}_{NaP} p(V_{n} - V_{Na} ) \end{aligned}$$

where $\bar{g}$ is the maximum conductance corresponding to each ionic current, their parameters were given in Table 1. $V_{Na}$, $V_{Ca}$, $V_{K}$, $V_{h}$, $V_{T}$ and $V_{L}$ are the reversal potential of ion channels and their parameters were given in Table 2. The calcium reversal potential varies with time [32]. $m$, $h$, $c$, $n$, $a$, $h_{A}$, $l$, $m_{T}$ and $p$ are gating variables [30].

**Table 1:** Distribution of ionic channels in cells compartments

Conductance	Dendrites	Initial segment	Narrow region	SOCB	Soma	Axon
$\overline{g}_{Na}$	25	150	200	400	80	70
$\overline{g}_{Ca}$	2	1.5	–	–	1.5	–
$\overline{g}_{K}$	12	18	18	–	18	18
$\overline{g}_{K,A}$	36	54	–	–	54	–
$\overline{g}_{K,Ca}$	0.001	0.065	0.065	–	0.065	70
$\overline{g}_{L}$	0.12	0.12	0.12	0.12	0.12	0.2
$\overline{g}_{h}$	5 × 10^−5^	5 × 10^−5^	5 × 10^−5^	–	5 × 10^−5^	5 × 10^−5^
$\overline{g}_{NaP}$	5 × 10^−5^	0.25 × 10^−5^	0.25 × 10^−5^	0.25 × 10^−5^	5 × 10^−5^	0.25 × 10^−5^
$\overline{g}_{T}$	25 × 10^−4^	5 × 10^−4^	5 × 10^−4^	5 × 10^−4^	5 × 10^−4^	5 × 10^−4^

All conductances are in units of ms/cm^2^

**Table 2:** The reversal potentials of the ion channels

Parameter name	Symbol	Value (mV)
Potassium reversal potential	*V*_*K*_	−70
Leak reversal potential	*V*_*L*_	−60
Hyperpolarization-activated reversal potential	*V*_*h*_	0
LVA calcium reversal potential	*V*_*T*_	120
Sodium reversal potential	*V*_*Na*_	35
Calcium reversal potential	*V*_*Ca*_	≈120 at rest

These model equations were numerically integrated in MATLAB R2010a using the Crank–Nicolson method at a fixed step size of 0.05 ms.

The extracellular stimulation in the model is monopolar stimulation. The stimulating electrode was modeled as a point electrode and fixed on 30 μm above the axon and horizontal distance 80 μm from the center of soma in this RGC model [33–35]. It takes time for the action potential to propagate along the axon to the distal axon. To better reflect the stimulus–response coherence, the recording site in the initial segment where the cell fired initially was the 15 μm distance from the center of the soma, as shown in Fig. 1.

### Stimulating current

**Fig. 2:**
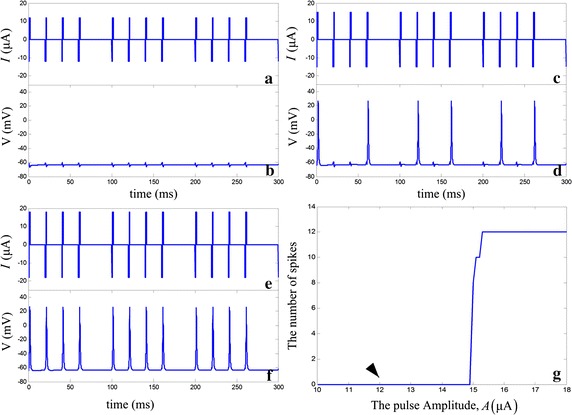
A time series of membrane potentials and stimuli of the noiseless RGC model. **a**, **b** The stimulating current and membrane potentials in *A* = 12 μA, **c**, **d** the stimulating current and membrane potentials in *A* = 15 μA, **e**, **f** the stimulating current and membrane potentials in *A* = 18 μA. **g** The *black arrow* points to the current amplitude of the subthreshold signal we take

The stimulating current consists of the subthreshold signal and the noise current. The subthreshold signal in the RGC model was biphasic rectangular current pulses without interphase gap. The pulse width generally applied to retinal implants stimulation was 0.05–1 ms and clinically retinal implants typically used stimulus pulses was the order of 1 ms [36]. Sequences of pulses at frequencies ≤250 Hz were used to elicit one spike per pulse [37]. The efficacy of electrical stimulation was higher for small number of pulses in a train and delivering the pulse sequence at a small rate [38]. Here the cathodic and anodic phase duration were 1 ms, 50 Hz and the waveforms were presented in the form of sequences of four biphasic rectangular pulses at a frequency 10 Hz, as shown in Fig. 2a.

Stochastic biphasic pulse sequences as noises were used to achieve SR, which were made up of a series of rectangular current pulses. The positive and negative phase duration were equal and their total pulse width was represented by $w$. $T$ denoted the interval between the start of two adjacent pulses. ${\text{T}}$ and $w$ were extracted from separate uniform distributions independently and changed in a dynamic range respectively. ${\text{T}}$ varied between $b_{w}$ and $b_{T}$ and $w$ varied between $a_{w}$ and $b_{w}$. The amplitude of each pulse in the biphasic pulse sequences was indicated by $a$, as shown in Fig. 4a. $a$ is constant for each stimulus and calculated as follows [24]

4 $$a = E\left[ {A_{rms} } \right]((b_{w} + a_{w} )/(b_{w} + b_{T} ))^{ - 1/2}$$

where $E\left[ {A_{rms} } \right]$ is the RMS amplitude of the stochastic biphasic pulse sequences.

### Characterizing SR-type behavior

Collins et al. proposed a method for characterizing SR-type behavior in excitable systems with aperiodic inputs: the power norm [39]. The power norm reflects the coherence between input stimulus and system response. Recently a measure related to the power norm was used [24]:

5 $$C_{1} = \frac{{\frac{1}{N}\sum\limits_{k = 0}^{k = N} {S_{k} R_{k} } }}{{RMS\left[ S \right] \cdot RMS\left[ {R - \frac{1}{N}\sum\limits_{k = 0}^{k = N} {R_{k} } } \right]}}$$

$$RMS\left[ x \right] = \left( {\frac{1}{N}\sum\limits_{k = 1}^{k = N} {x_{k}^{2} } } \right)^{{{1 \mathord{\left/ {\vphantom {1 2}} \right. \kern-0pt} 2}}}$$

where *S* is the input signal with zero-mean. *R* is an indicator variable that represents whether or not there is any action potential at sample *k*, of which there are *N* for each simulation. *R* is composed of a series of zero and one equal in length to the input signal. In the 1 ms windows centered at the time of each action potential *R* are set to a value of one and the rest are zero. Action potentials were identified as peaks in the voltage that exceeded 20 mV.

### Implementation of the GA

In terms of producing the minimum power consumption and charge of the stimulus waveform and evoking the action potential, the noise parameters ($b_{w}$, $b_{T}$, $E\left[ {A_{rms} } \right]$) were optimized by using the GA. Each noise parameter was represented by a gene.

The charge balance of stimulating pulse was a necessary condition of electrical stimulation. Net charge injected to stimulate tissue may cause tissue damage and electrode corrosion [40]. In order to ensure the charge balance, the same noises were added to cathode and anode phase. Furthermore, the cathode phase of waveform contributed to activation of the RGC, so the noise parameters added to cathode phase were optimized. The energy consumption of each electrical stimulation waveform and the amount of charge during a cathode phase were calculated as follows [41]:

6 $$E = \int_{0}^{PW} {I^{2} \left( t \right)} Z\left( t \right)dt = dt*\sum\limits_{n = 0}^{N} {I_{n}^{2} Z_{n} }$$

7 $$Q = \int_{0}^{{T_{c} }} {I\left( t \right)} dt = dt*\sum\limits_{n = 0}^{N} {I_{n} }$$

where *PW* is the duration of the pulse waveform. $I$ is the stimulating current. $Z$ is the load impedance and equal to 1 kΩ. $dt$ is the time step of discretizations. $N$ is the number of discretizations in the duration of the pulse waveform. $T_{c}$ is the duration of cathode phase. The cost function of each waveform, $F1$, equaled $E$ plus $Q$ and a considerable penalty, $P1$:

8 $$F1 = E + Q + P1$$

$P1$ is 0 if the waveform elicited an action potential, and 1 nJ if it did not.

The number of stimulation waveforms per generation of the GA is 100. The values of the genes of the waveforms of the first generation were selected at a stochastic uniform distribution between 0 and 1. The GA was performed for eight times independently, each for 200 generations and with different initial populations. The mean and standard error of the optimal noise parameters and the minimum cost values are calculated.

## Results

### SR in the RGC model

Noiseless stimulating current was applied to the model and its threshold value was determined by varying current amplitude. The threshold value was defined as the minimum current magnitude required to elicit an action potential. The pulse amplitude of the noiseless stimulating current was presented by *A*. Figure 2 depicts dynamical responses of RGC model under different current amplitudes. Apparently, for *A* = 12 μA, the signal is too weak to excite the RGC in Fig. 2b. As the current amplitude is increased, the RGC is excited to output spike trains (Fig. 2d, f). When the current amplitude is 15.3 μA, there is a response throughout each input pulse, so the threshold of the stimulation pulse was 15.3 μA, as shown in Fig. 2g.

**Fig. 3:**
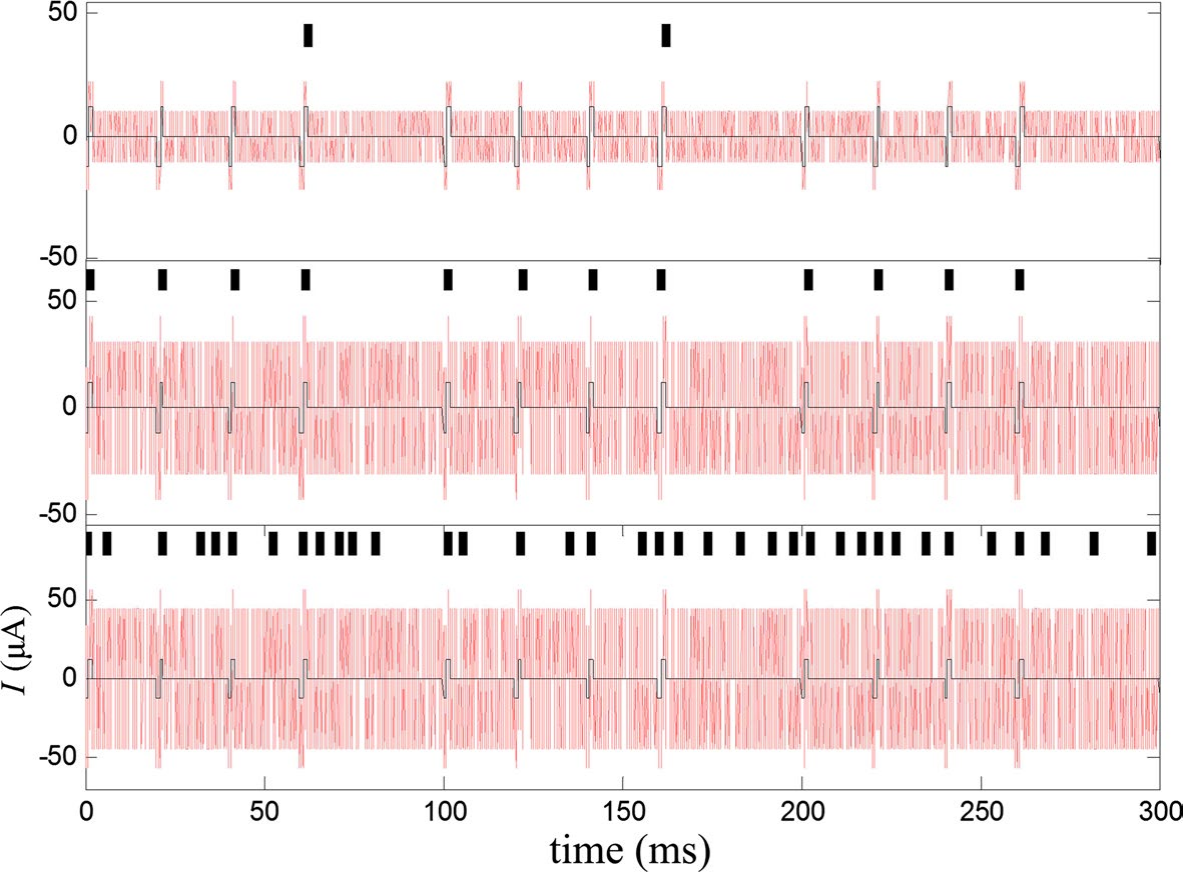
The spike trains for various RMS amplitude of the stochastic biphasic pulse trains. Noise (*red traces*) added to a subthreshold signal (*black traces*) and the spike trains (raster above each trace). From *top* to *bottom*: the RMS amplitudes of noise are 6, 18, 30 μA and *a*_*w*_, *b*_*w*_ and *b*_*T*_ are fixed at 0.01, 0.1 and 0.45. The power norm *C*_1_ that correspond to each stimulus are 0.11, 0.53, 0.13 respectively

Stimulating current with the stochastic biphasic pulse sequences was applied to the RGC model. The subthreshold signal consisted of a series of stimulus pulses, less than 80 percent of threshold value (12 μA). Then the RGC model response was studied by varying the RMS amplitudes of the stochastic biphasic pulse sequences and $a_{w}$, $b_{w}$ and $b_{T}$ are fixed. Figure 3 displays the model responses under three RMS amplitudes of noises. At low RMS amplitudes the model generated few spikes and at higher RMS amplitudes spikes were almost synchronous with the input stimulus pulses; however, with the RMS amplitudes further increased, the model generated spikes whether or not stimulus was present.

### The influence of noise parameters on SR

The shape of the stochastic biphasic pulse sequences as noise was determined by the interval between the start of two adjacent pulses, T, each pulse width, $w$, and the pulse amplitude, $a$. The pulse amplitude was calculated as Eq. (4).

**Fig. 4:**
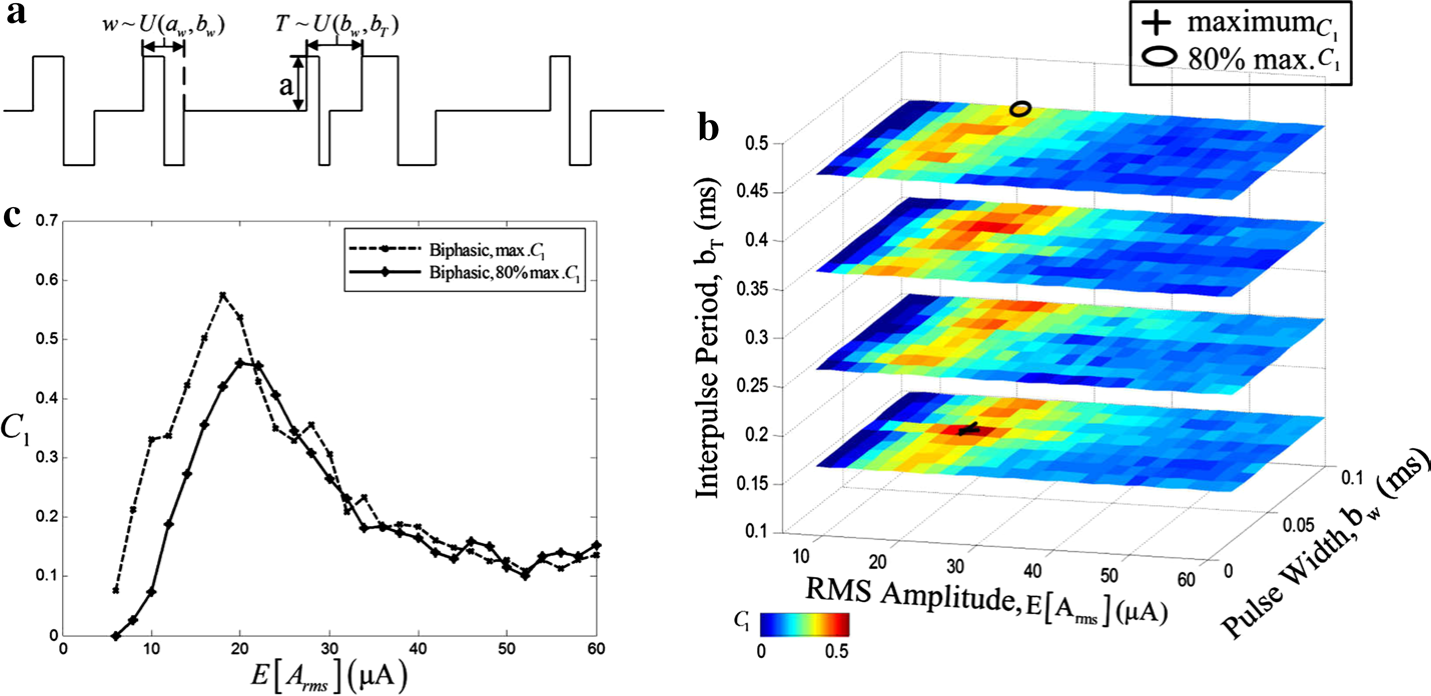
SR is generated by stochastic biphasic pulse trains in the RGC model. **a** The stochastic biphasic pulse train used as noises. **b** The corresponding power norm in the parameter spaces. The *a*_*w*_ parameter is a constant 0.01. The *black cross* in figure is the location of the maximum. The *black oval* represents about 80% max. **c** The curves of *C*_1_ versus *E*[*A*_*rms*_]

Traditionally, the way that found the optimal power norm $C_{1}$ for SR applications only was to vary the perturbation intensity of noises, that is, the RMS amplitude, $E\left[ {A_{rms} } \right]$. However, the stochastic biphasic pulse sequences were decided by four parameters, $a_{w}$, $b_{w}$, $b_{T}$ and $E\left[ {A_{rms} } \right]$, so a 4-dimensional optimization could be performed. In this paper, a 3-dimensional optimization was performed and one parameter, $a_{w} = 0.01\;{\text{ms}}$, was fixed. The power norms $C_{1}$ were calculated in a parameter space, $0.01\;{\text{ms}} < b_{w} < 0.15\;{\text{ms}}$, $0.15\;{\text{ms}} \le b_{T} < 0.5\;{\text{ms}}$. The results of $C_{1}$ in the parameter space are plotted in Fig. 4b. $C_{1}$ corresponding to each parameter combination was the average value of five 300 ms stimulations in response to the stimulating currents.

Figure 4b shows the performance of $C_{1}$ as the function of $b_{w}$, $b_{T}$ and $E\left[ {A_{rms} } \right]$ with a reduced space which included the changed trend. The maximum $C_{1}$ indicated by black cross in Fig. 4b is 0.5744. High $C_{1}$ appeared regardless of long or short interval between the start of two adjacent pulses. However the large pulse width resulted in the higher $C_{1}$ and the RMS amplitudes corresponding to near-optimal $C_{1}$ values were less than 30 μA. Figure 4c shows the change of $C_{1}$ varying the RMS perturbation amplitude. The values of $C_{1}$ shows the inverted U-like graph. For each stimulus, there existed an optimal value of the RMS amplitude corresponding to the maximum $C_{1}$.

### Optimization of noise parameters

**Fig. 5:**
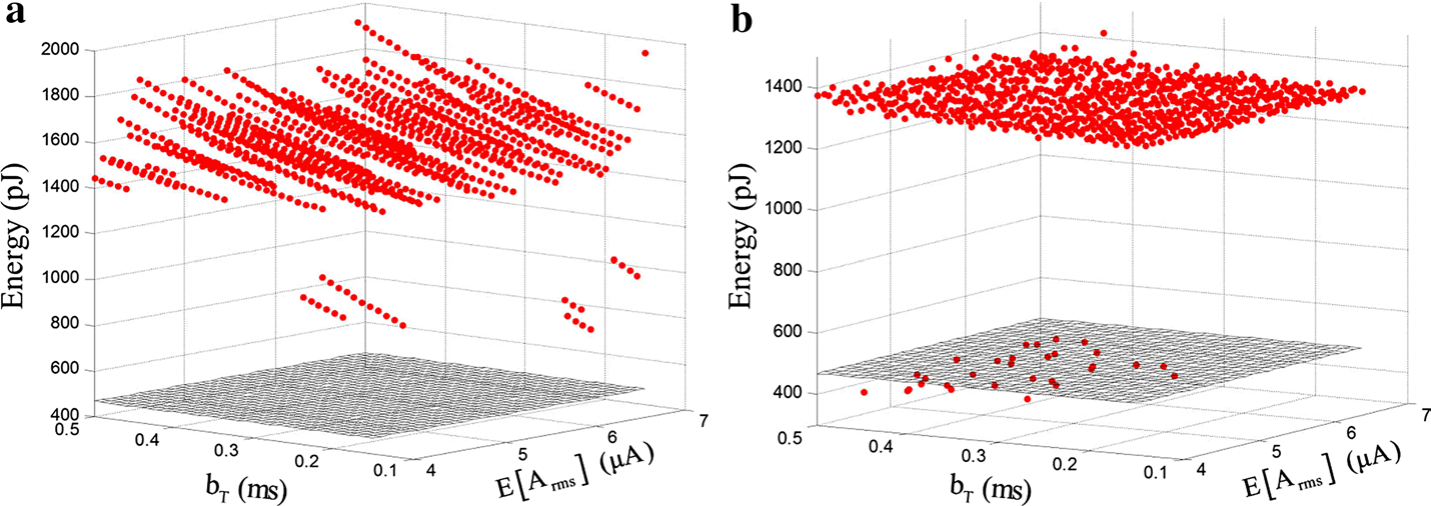
The energy consumption of a single stimulus pulse. **a** The energy of a single stimulus pulse used to generate SR (*red dot*). **b** The energy of the adjusted stimulation pulse (*red dot*). *Gray* grid plane is the energy consumption of threshold

To generate SR, stochastic biphasic pulse sequences were added to the subthreshold signal, but additive noises were companied by additional energy consumption. The energy consumption of a single 10 ms stimulation pulse was calculated and compared with the energy of threshold level. As shown in Fig. 5a, the energy consumption of a single stimulus waveform is more than that of threshold level. In order to reduce the energy consumption caused by noises, the strategy of adding noises was adjusted and the noise only was added to the cathode and anode phase of the subthreshold signal. Figure 5b shows that the energy changes under different noise parameters after adjusting. The energy consumption of the stimulation pulse includes the penalty value. If the stimulation pulse can’t elicit an action potential, the penalty value equals 1 nJ. It was possible that the energy consumption of the adjusted stimulation pulse was less than that of threshold. Figure 5 is the result of local noise parameters, wherein $b_{w} = 0.055\;{\text{ms}}$.

**Fig. 6:**
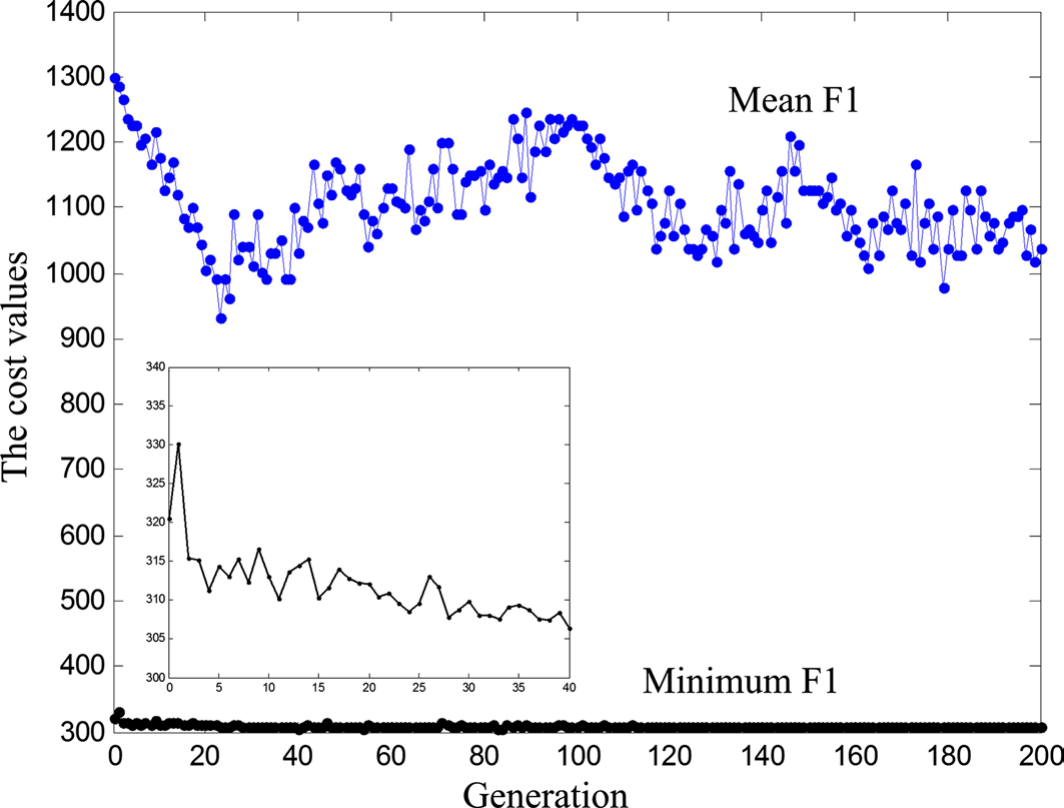
The mean and minimum cost values across 200 generations. The local magnified image of 0–40 generations is embedded in the figure

**Fig. 7:**
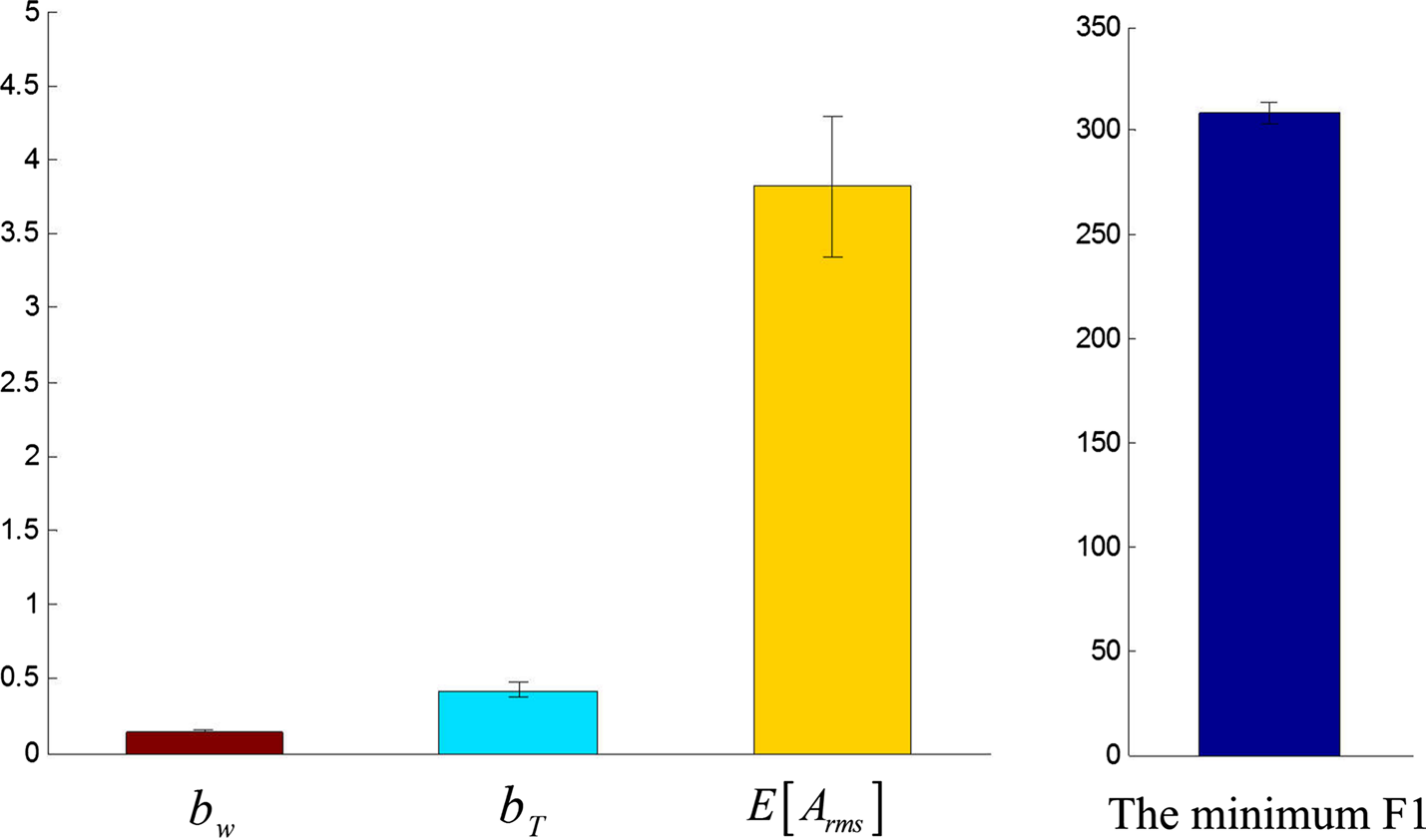
The mean and standard error of optimal noise parameters and the cost values

The optimal noise parameters corresponding to the smaller energy consumption was found by performing a GA. The optimal ranges of noise parameters, $b_{w}$, $b_{T}$, were as above mentioned in this section. The smaller values of $E\left[ {A_{rms} } \right]$ were selected and varied between 1 and 5 μA. With the progress of the GA, the minimum and mean cost values decreased gradually, until they were saturated. The one of results is shown in Fig. 6. In the progress of optimization, the mean cost value varied between 900 and 1300 pJ irregularly. The minimum cost value first sharply and then slowly tended to saturation. The local magnified image in Fig. 6 represents the downtrend obviously. After the GA was run for eight times, 200 generations per time, the mean of minimum F1 was 308.6690 pJ and the mean of optimal noise parameters were *b*_*w*_ = 0.1449 ms, *b*_*T*_ = 0.4204 ms, $E\left[ {A_{rms} } \right]$ = 3.8184 μA, as shown in Fig. 7. The energy consumption and the charges of threshold level are 468.18 pJ, 15.30 nC. Compared with the threshold level, the sum of energy consumption and charges of stimulation waveform after optimization are reduced by 36.2%.

**Fig. 8:**
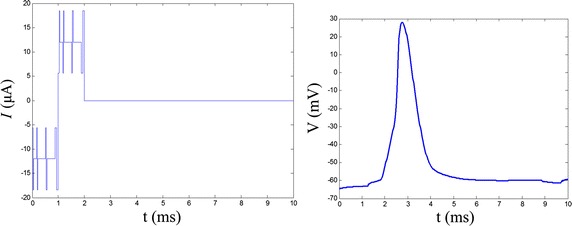
The stimulation waveform corresponding to the minimum energy consumption and the action potential

Noise parameters are fixed for the mean of optimal noise parameters and the subthreshold signal with these noises were used to stimulate the RGC model. The energy consumption and charges of the stimulation waveform were calculated. Repeating the stimulation 15 times, the average energy consumption and charges were 314.8174 pJ, 11.9281 nC, which are reduced by 32.8 and 22.0% relative to the threshold level. The maximum values were 327.4733 pJ, 11.9964 nC and the minimum values were 285.4879 pJ, 11.6608 nC which are reduced by 39.0 and 23.8%, respectively. The stimulation waveform corresponding to the minimum energy consumption and RGC response are shown in Fig. 8.

## Discussion

Our study found that the power norm of versus the RMS amplitude of noise is the inverted U-like curve characterized by maximal power norm at a specific noise intensity value. It is consistent with the essential feature of SR phenomenon [42]. This shows SR exists in the RGC. When certain intensity of noise is added to the subthreshold signal, it achieved the same efficacy as that of threshold signal (Fig. 3). This may be helpful to increase brightness and the dynamic range of phosphene so that some electrodes are allowed to elicit phosphene that are previously unable to do so due to electrochemical safety limit restrictions and enhance the detection of visual information in retina [43, 44]. The RGCs themselves are present in a noisy environment and the addition of artificial noise accords with the actual physiological conditions.

In this study, noise is added to the subthreshold signal is used as a way to reduce stimulation threshold and energy. In this method, the noise parameters have an effect on the energy consumption of a single stimulus waveform (Fig. 5). After optimizing the noise parameters using a GA, the energy consumption of the adjusted stimulation pulse is lower than that of threshold level. Hadjinicolaou et al. optimize interphase interval and phase duration led to a median charge saving of 14 and 20% respectively [38]. Here, the energy consumption and the charges of the single stimulation waveform are reduced by 39.0 and 23.8% relative to that of the threshold level. Additionally, compared to the method of reducing threshold and the energy by minimizing the distance between the multielectrode array and retina [11], our study avoids increasing the difficulty of surgical techniques. Changing the stimulus waveform is the conventional way of reducing threshold and the energy [26], but this method increases the difficulty of circuit implementation. Both subthreshold signal and noises in our study are biphasic rectangular pulses and it is easier to implement. These make it feasible to reduce stimulation threshold, energy consumption and charges without altering bipolar rectangular pulse stimulation.

Epiretinal prosthesis is implanted in the retina for a long time and retinal neurons tolerate long-term electrical stimulation. The energy consumption was an important factor for evaluation of stimulation parameters [43]. In this study, only adding noises to the cathode and anode phase of the subthreshold signal that can elicit action potential further reduces energy consumption.

SR can enhance the detection and transmission of subthreshold stimuli in neurons and has been applied to some prostheses. SR could enhance modulation sensitivity in cochlear implant listeners and decrease the threshold to an information-bearing signal [20, 45]. The temporal representation of speech cues can be improved by adding optimal noise to cochlear implant signals [46]. Mechanical noise introduced into the feet via vibrating insoles improved balance in standing position [47]. To more closely replicate natural breathing, random noise was applied to the operation of the artificial ventilator [22]. This study extends the application of SR in neural prosthetics and provides further support for Danziger and Grill’s research. Additionally, our study applied the extracellular stimulation which is more similar to the implantation of neural prostheses.

Our studies show that the energy consumption and charges of the stimulation waveform can be reduced by adding optimal noises to subthreshold signal and these may be helpful for improving the performance of epiretinal prosthesis. The ability to control parameters precisely is the main advantages of a computational simulation approach over experimental approaches. Nonetheless, this multi-compartments model here does not represent all RGCs on account of different types and morphologies of RGCs in retina. Previous studies have shown the effect of RGC morphology on its electrophysiological responses [30]. It is worth further studying that how RGC morphology influences optimization of noise parameters. Besides, if it is applied to the actual, our research needs further experimental verification.

## Conclusions

The results show that SR exists in the RGC and can enhance the response of RGC to a subthreshold signal. The stochastic pulse sequences can be used to generate and tune SR in the RGC. After adjusting the strategy of adding noise and optimizing the noise parameters, the energy consumption and charges of the stimulation waveform are reduced largely. These demonstrate that it is feasible to reduce the stimulation threshold, the energy consumption and charges by adding noise to the subthreshold signal. Reducing threshold will help to expand the scope of electrode-induced phosphene and increase brightness. With the energy consumption and charges decreasing, the lifetime of epiretinal prosthetics can be prolonged and the tissue damage is reduced, which will help the development of retinal prosthesis.

## Abbreviations

RGCs: retinal ganglion cells
SR: stochastic resonance
GA: genetic algorithm
RP: retinitis pigmentosa
AMD: age-related macular degeneration
SOCB: sodium channel band
FCM: Fohlmeister–Colman–Miller
LVA: low voltage activated
